# Extracorporeal Rescue for Early and Late Graft Failure after Cardiac Transplantation: Short Result and Long-Term Followup

**DOI:** 10.1155/2013/364236

**Published:** 2013-10-08

**Authors:** Nai-Kuan Chou, Nai-Hsin Chi, Hsi-Yu Yu, Jou-Wei Lin, Chih-Hsien Wang, Shoei-Shen Wang, Yih-Sharng Chen

**Affiliations:** Department of Cardiovascular Surgery, National Taiwan University Hospital, Taipei 100, Taiwan

## Abstract

*Objectives*. Graft failure after heart transplantation led to poor outcomes. We tried to analyze the outcomes of extracorporeal membrane oxygenation (ECMO) rescue in graft survival after transplantation. *Methods*. A retrospective review of 385 consecutive heart transplants revealed 46 patients of graft failure requiring ECMO rescue (48 episodes). The pretransplant and ECMO-related variables were evaluated. *Results*. The median age was 37.7 ± 18.8 years, and the median support time was 155 ± 145 hours. Success rate was 47.9% (23/48). Pretransplant ECMO use was 25% (12/48) and they had 58.3% mortality. The success rate in “early” graft failures was 51.4% (18/35) and 50% for “late” graft failure. The ischemic time with graft failure (178 ± 70 min) was significantly longer than that without graft failure. Preoperative status and the longer ischemic time may be the major factors for failure. Long-term 5-year survival demonstrated significant survival difference between graft failure and nongraft failure. No survival difference was shown between “early” and “late” graft failure. *Conclusions*. Graft failure still carried high mortality if advanced circulatory support was required. Early graft failure and late graft failure had similar outcomes. Further investigation of the risk factors shows that ECMO does play a role of rescue in catastrophic conditions.

## 1. Introduction

Graft failure after cardiac transplantation remains a significant source of mortality, especially for the primary graft failure [1, 2]. It is defined as severe dysfunction of cardiac allograft without any anatomic cause. Also, it is characterized by hypotension, high filling pressure, and refractory low output [3]. The graft failure after heart transplantation may present a wide spectrum of clinically apparent allograft dysfunction, including right heart dysfunction without evidence of a high afterload, isolated left ventricular dysfunction, and even biventricular dysfunction [2]. The cause of graft failure may be related to immunological issues, insufficient myocardial protection, pulmonary hypertension, or possible underlying sepsis [1, 3]. Sometimes, it is difficult to clearly differentiate the real etiologies because there might be mixtures of the causes described above. Emergent retransplantation for this status had very poor results [4, 5]. They usually required mechanical circulatory support more than just a intraaortic balloon pump (IABP), but the mortality still remains quite high [1, 2, 5–7].

We have documented that extracorporeal membrane oxygenation (ECMO) could be applied in several situations, including myocarditis [8], postcardiotomy shock [9], and other conditions [10]. We applied ECMO in posttransplant status, either in the early or late stage for their critical condition since 1995; we tried to evaluate the role and result of ECMO in resuscitating this particular patient group. 

## 2. Material and Methods 

The present study was approved by the Institutional Review Board of Investigation. 

### 2.1. Patients

Between 1987 (i.e., our institute began the first heart transplantation) and April of 2010, a total of 385 consecutive patients underwent cardiac transplantation. We retrospectively reviewed our experience of applying ECMO after transplantation and followed these patients until 2012. The patients were either early or late phase and due to primary graft failure or late rejection were leading towards myocardial dysfunction. The present study described and analyzed the result of ECMO application to rescue graft failure after heart transplantation refractory to medical therapy and intraaortic balloon pump (IABP) in the posttransplant period. According to the definition of graft failure after heart transplantation given elsewhere [1, 5], the graft failure after heart transplantation was made by diagnosis with exclusion of possible anatomical factors, treatable pulmonary hypertension, and hypoxemia. We briefly categorized the graft failure after heart transplantation into “early” and “late” graft failure after heart transplantation. The “late” graft failure after heart transplantation was usually considered ECMO application > 60 days after cardiac transplantation. The “early” graft failure after heart transplantation was considered ECMO application within 7 days after transplantation. We also specified the subgroup with “immediate” graft failure after heart transplantation, ECMO support since operation room (OR), which might be usually considered to have poorer outcomes. An ECMO episode was only counted once for the first setup in the same admission. Different times of ECMO support in different admissions were considered to be different episodes. 

### 2.2. Immunosuppressant Protocol

A “triple immunosuppression” regimen basically consisting of cyclosporine A (ciclosporin A), methylpredisolones, and azathioprine was utilized in the immediate post-transplant period. Rabbit antithymocyte globulin immunoinduction therapy was administered during the first 5 days of induction. Cyclosporine started 5 days after transplantation or after recovery of renal function depending on patient's clinical status. For clinical evaluation of cardiac graft rejection, transvenous endomyocardial biopsy was performed three to four times in the first month and then every 3 months in the first year. The dosage of immunosuppressants was adjusted by the results of endomyocardial biopsies. Tacrolimus (FK-506) and mycophenolate mofetil (Cellcept) were used for recurrent rejection or severe adverse reactions to cyclosporine and azathioprine. The immunosuppressant agent would be reduced when infection was highly suspected. 

### 2.3. Management of ECMO

The whole ECMO apparatus, including centrifugal pump and oxygenator (Medtronic Inc. Anaheim, CA) was primed with normal saline alone due to the emergent situation. The protocol and procedure had been described elsewhere [11]. Cannulation was performed with a modified open Seldinger method. The pressure in the superficial femoral artery was measured after cannulation. If the mean pressure was below 50 mmHg, a perfusion catheter (8.5 Fr, Super Arrow-Flex central venous catheter) was inserted distally [12].

The ECMO circuit consisted of a centrifugal pump, a hollow fiber microporous membrane oxygenator, and percutaneous thin-wall cannula (Medtronic Inc., Anaheim, CA), all of which were coated with a heparin-bound Carmeda Bioactive surface. VA ECMO was routinely instituted from the groin area. The 800 mL ECMO priming solution contained 1600 U heparin; the tubing sets in our ECMO circuit were heparin-bound, and expected that the duration of ECMO support. When a small femoral artery was found after exploration of the femoral vessels and the distal leg perfusion was not adequate after arterial cannulation, a small additional tube connected by a Y-adapter was inserted to the distal leg to prevent distal leg ischemia. 

 In the event of extension of the duration of ECMO support from temporary to prolonged use, low-dose heparin was administered to keep the activated clotting time at 160 to 180 seconds in order to prevent ECMO-related hemolysis or thrombotic complications. 

 When the ECMO-related hemolysis or plasma leaked from the oxygenator, then the entire circuit was changed.

Hemodilution after the hook-up of ECMO to the patients was corrected by packed red blood cells transfusion. Hematocrit was kept between 30% and 35%. Lower hematocrit compromised oxygen delivery, but higher hematocrit increased complications of clot formation and hemolysis by centrifugal pump.

Continuous monitoring of postoxygenator blood oxygenation saturation provided an indicator for the gas exchange function of the oxygenator. Systemic heparinization was not needed on the first day of ECMO support; there would be higher risk of bleeding immediately after the heart transplantation operation. Whatever happens, heparin infusion would not be used until the bleeding was controlled, which usually took 1 to 2 days. Once bleeding had decreased, we would begin heparin drip and keep the activated clotting time between 160 and 180 seconds. We changed the ECMO apparatus when oxygenator dysfunction, clot formation, or hemolysis were found. Symptoms and signs of low cardiac output were usually resolved after the ECMO support was initiated, and catecholamine infusion could be tapered accordingly. Arterial pulse pressure wave contour, serial echocardiography, and blood oxygenation saturation in the preoxygenator circuit were used to monitor the recovery of cardiac allograft. 

 From our experience, we were able to achieve initial cardiac recovery signs based on insufficient information about the patients on ECMO for the arterial pulse contour.

If hemodynamics could be well maintained by reduced ECMO blood flow at 0.5 L/min for 1 hour, perhaps ECMO should also be removed at bedside. Anyhow, the wound was primarily repaired.

### 2.4. Data Collection and Analysis

We treated each ECMO course for each episode. Each ECMO course was defined from setup to hospital discharge or death in hospital. We counted for the “failure” or “success” for each ECMO episode. “Failure” was defined as failed in surviving hospital discharge; even they might be weaned ECMO successfully and die in hospital because of complications. “Success” was defined as successful weaning ECMO and surviving to hospital discharge. Donor-, surgery-, and ECMO-related variables were evaluated for association among “failure” and “success.” Continuous variables were expressed as mean ± standard deviation, and means were compared by independent sample Student's *t*-test. Nominal variables were expressed as percentages and analyzed by the *χ* ^2^ test. Statistical significance was assumed at a *P* value of less than 0.05. The Kaplan-Meier survival curve was performed according to their long-term follow-up data. 

## 3. Results

There were a total of 46 patients (37 males, 11 females) with 48 episodes of ECMO applications for graft failure after heart transplantation who were recruited in the present retrospective study. The mean age was in the range 37.7 ± 18.8 years. In the same period, there were 387 consecutive patients that underwent cardiac transplantation in our institution. There were 12 episodes requiring pretransplantation ECMO support (25%), and “early” graft failure after heart transplantation were 35 episodes (72.9%), in which 30 episodes were “immediate” graft failure after heart transplantation (62.5% in whole group, 85.7% in “early” graft failure after heart transplantation). Five episodes of ECMO setup occurred in intensive care unit within 7 days after transplantation. ECMO was set up in the rest of 13 episodes > 7 days after transplantation, and 10 of them were categorized as “late” graft failure after heart transplantation (>60 days after transplantation). 

One male patient received 3 times of ECMO support after transplantation. He was a case of congenital corrected transposition of great arteries and received conventional repair when he was 2 years old. He developed systemic ventricular failure 9 years after repair. He also received emergent ECMO support under resuscitation due to the deteriorated congestive heart failure and sudden cardiac arrest. He received a heart transplantation after 68-hour support. graft failure after heart transplantation developed, and the ECMO was supported for additional 26 hours with successful decannulation and discharge from hospital. However, acute graft dysfunction happened 2 years after discharge. Therefore, he had to received another installation of ECMO for 132-hour support because of rejection leading to unstable hemodynamic conditions. He was discharged again uneventfully. Unfortunately, progressive graft dysfunction persisted due to vasculopathy leading to collapse at the fourth year after transplantation. He received ECMO for the third time and was listed for retransplantation due to a collapse. He underwent re-transplantation and early graft failure after heart transplantation occurred. ECMO was extended for an additional 173 hours with successful wean-off, but he still expired 63 days after the retransplantation due to multiple organ failure and sepsis.

The overall success rate of graft failure after heart transplantation was 47.9% (23 episodes surviving to discharge in 48 episodes). The success rate was 51.4% (18/35) in “early” graft failure after heart transplantation and 50% (5/10) in “late” graft failure after heart transplantation. The subgroup of “immediate” ECMO had 50% success rate as well (Table 1). Five episodes developed in intensive unit after transplantation within 7 days, and 4 episodes (80% success rate) were successfully survived to discharge. But the success rate was low (0/3, *n* = 3) in ECMO support between 7 and 60 days after transplantation. The data of the present study, “early,” “immediate,” and “late” graft failure after heart transplantation were demonstrated in Table 1.

**Figure 1:**
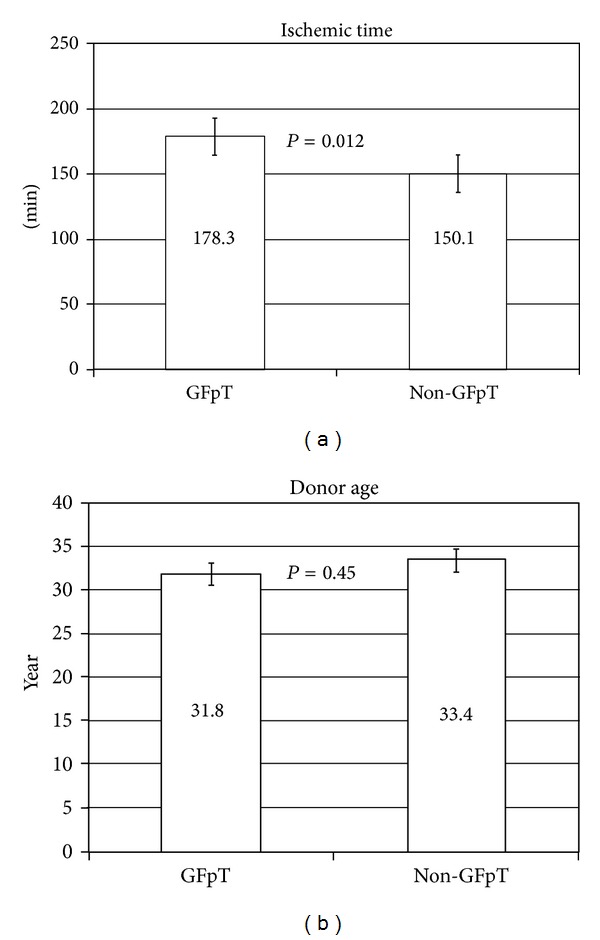
(a) Comparison of ischemic time between graft failure after heart transplantation (graft failure after transplantation) and nongraft failure after heart transplantation (*P* = 0.0012). Ischemic time in graft failure after heart transplantation was 178.3 ± 70.4 min (median 169 min); 47.9% of them were over 180 min. Ischemic time in nongraft failure after heart transplantation was 150.1 ± 64.9 min (median 132 min); (40 to 330 min), 32.9% of them were over 180 min. (b) Comparison of donor age and ischemic time between graft failure after heart transplantation versus nongraft failure after heart transplantation. Age: graft failure after heart transplantation 31.8 ± 14.1 yr (median 33), nongraft failure after heart transplantation 33.4 ± 12.8 yr (median 32), *P* > 0.05.

The ECMO duration was 155 ± 145 hours, and IABP were applied in 31.3% of the study group. The ischemic time (Figure 1(a)) and donor age (Figure 1(b)) of graft failure after heart transplantation was 178.3 ± 70.4 min (median 169) and 31.8 ± 14.1 years (median age was 33). We compared the data with nongraft failure after heart transplantation group (*n* = 339), and it revealed significant difference in donor ischemic time (Figure 1(a)). 

**Figure 2:**
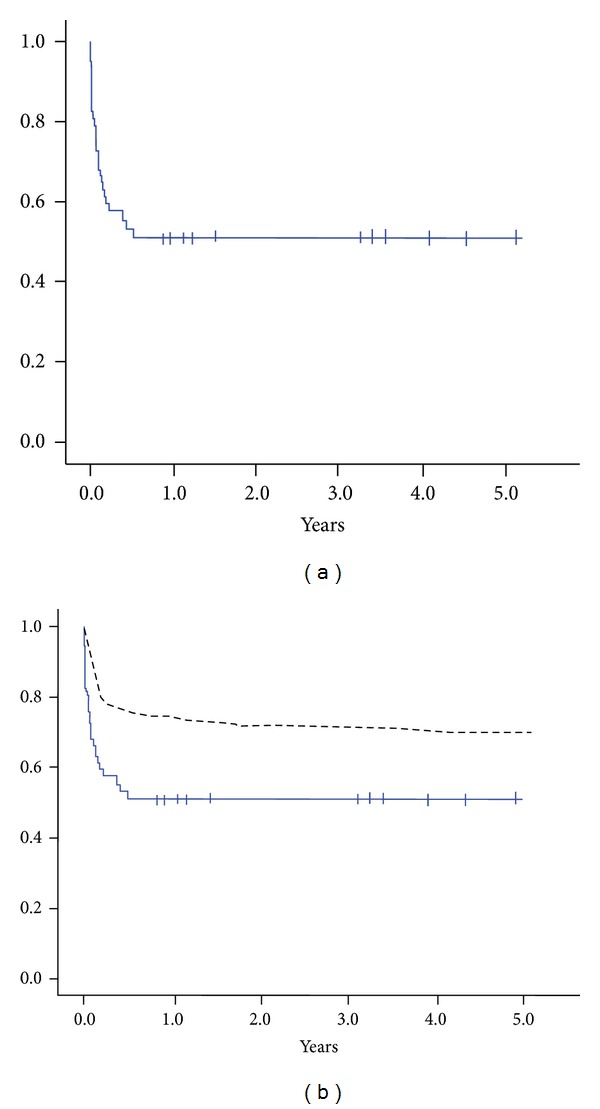
(a) The five-year survival curve of the study group (graft failure after heart transplantation). (b) Comparison of the nongraft failure after heart transplantation and Graft failure after heart transplantation survival curve. Blue: graft failure after heart transplantation, black dash line: nongraft failure after heart transplantation survival curve of our center.

The 5-year survival curve was around 45% survival in 5-years (Figure 2(a)). The comparison with the survival curve of our center showed a significant difference during 5 year survival (Figure 2(b), black dash line, Log rank *P* < 0.01). 

**Figure 3:**
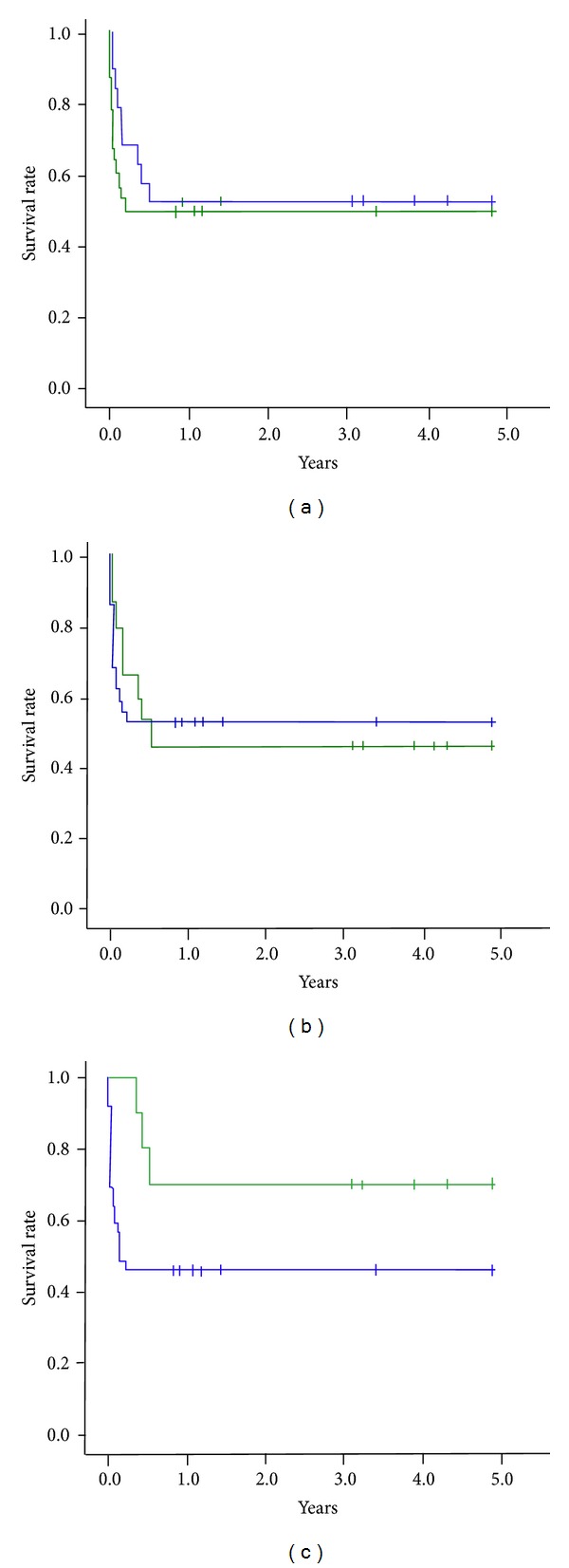
(a) The 5-year survival curve between immediate (green) versus nonimmediate (blue) graft failure after heart transplantation, log rank *P* = 0.548. (b) The 5-year survival curve between ECMO < 7 days (blue) versus ECMO > 7 days (green), Log rank *P* = 0.971. (c) 5-year survival curve between ECMO at <60 days (blue) versus ECMO at >60 days (green), log rank *P* = 0.103.

The 5-year survival curve between “immediate” graft failure after heart transplantation and “nonimmediate” graft failure after heart transplantation was demonstrated in Figure 3(a). The “early” graft failure after heart transplantation (ECMO < 7 days) also did not show a poorer survival than those with ECMO > 7 days after transplantation (Figure 3(b)). 

The survival curve comparison between “late” graft failure after heart transplantation (ECMO at >60 days after transplantation) and those ECMO < 60 days also did not show any statistical difference (Figure 3(c)). The “late” graft failure after heart transplantation might be a different group of graft failure from the “early” graft failure after heart transplantation, which was mainly due to acute humeral rejection, vasculopathy, or sepsis. 

**Figure 4:**
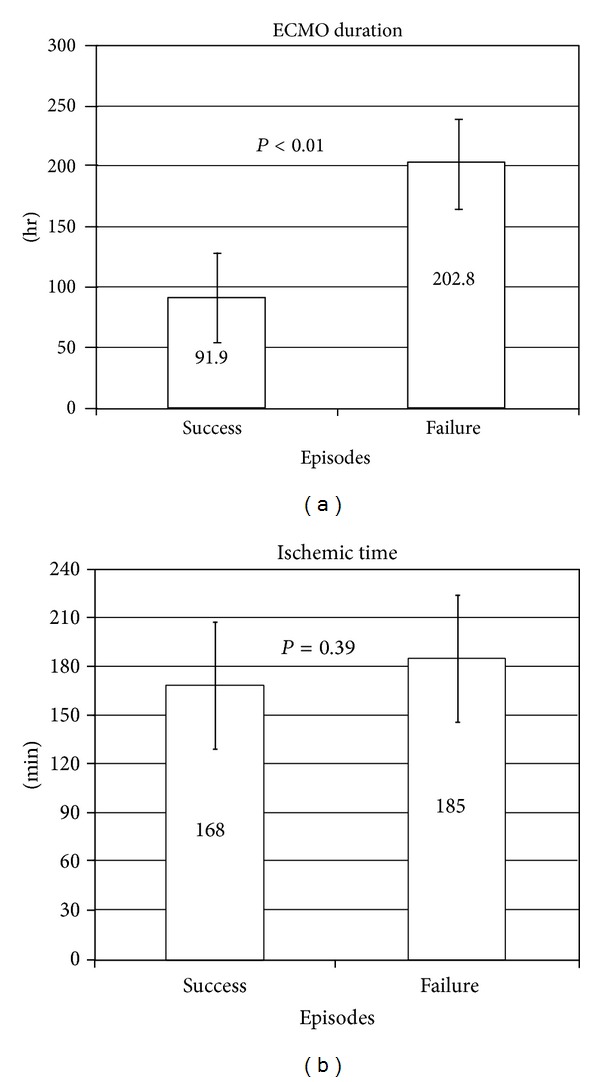
(a) Comparison of ECMO duration between success and failure episodes. ECMO duration is a significant risk factor for failure episodes. (b) Comparison of donor ischemic time between success and failure episodes. The donor ischemic time was not a risk factor for failure episodes.

We also examined the risk factors between the success and failure episodes. The only significant difference between the two episodes was the ECMO duration (91.9 ± 48.7 hr versus 202.8 ± 162.1 hr, *P* < 0.01, Figure 4(a)), which seemed reasonable since the failure episode tended to have longer support before expiration. The donor ischemic time did not show any difference between episodes (168 ± 72 min versus 185 ± 71 min, *P* = 0.39, Figure 4(b)).

## 4. Discussion

graft failure after heart transplantation requiring circulatory support is associated with significant mortality, not only in early graft failure after heart transplantation but also in late graft failure after heart transplantation. ECMO could work as a good rescue tool for these risky patients. The result was acceptable, Furthermore, it is improving than the previous literature [1, 5, 13]. It could be applied in the early stage after transplantation. It also acts as a rescue for those with acute rejection or acute developed sepsis. It offers enough safe evaluation periods for accurate diagnosis and proper treatment.

We previously, thought the “immediate” graft failure after heart transplantation might result in poor long-term outcome compared to that of “nonimmediate” graft failure after heart transplantation because the special subgroup was considered to have high mortality even when mechanical support was applied [2, 5, 13]. According to data of Table 1, “early” graft failure after heart transplantation might be considered to have longer ischemic time, poor myocardial preservation or poor recipient conditions since they had higher incidence of extracorporeal resuscitation (ECPR). But the result seemed to be unsupportive than what we had previously thought before. Our result may suggest that ECMO could work as a rescue tool for acute deterioration no matter what the cause is, and it offers a 50% survival rate.

As stated by the International Society of Heart and Lung Transplantation (ISHLT) report, early graft failure is a major cause of death in the perioperative period after transplantation [14]. Its incidence varied from 4 to 24% depending on what the definition was [2, 5, 15, 16]. It might define the need for mechanical support or requiring high-dose inotropic support. We only recruited and defined the patients with the most severe status who were supported by ECMO, and it tended to have poorer results than ever. The incidence of “immediate” graft failure after heart transplantation was 7.8%, and the “early” graft failure after heart transplantation was 9.1%. In spite of the critical conditions, our result was comparable to the data from D'Alessandro et al., France [16]. We had almost a 50% success rate, in which the outcome was much better than that supported with ventricular assist device or re-transplantation [4, 5]. We agree with the concept that ECMO should be considered as the treatment of choice for graft failure after heart transplantation [17]. For our study, we extended the ECMO application in “early” graft failure after heart transplantation to also include “late” episodes.

We tried to figure out any risk factors for failure but failed to demonstrate any specific factor related to failure except the ECMO duration. The donor ischemic time and pretransplant ECMO application were not significantly different between successful and unsuccessful graft failure after heart transplantation. Even for CPR, we could not identify it as a risk factor. It might be due to the limited case number or the efficient resuscitation and ECMO initiation in our center [10]. 

We identified a specific subgroup within the study with extremely high mortality (100%), graft failure after heart transplantation patients receiving ECMO between 7 and 60 days. We thought it might be related to the heterogenic cause, mixture of rejection, and sepsis in the period or was a statistical issue only. The conclusion remains as further investigation and data collection in the future.

The causes of graft failures after heart transplantation sometimes were difficult to determine. The major cause for “late” graft failure after heart transplantation group was considered to be acute rejection, chronic vasculopathy with deterioration, and septic episodes for the “late” graft failure after heart transplantation group. However, it was difficult to differentiate these causes. The uncertain diagnosed causes in “early” graft failure after heart transplantation group were quite common [11, 16, 18]. It might be considered as primary graft failure, right heart failure, acute rejection, sepsis, pulmonary hypertension episodes, or unknown causes. Since it was difficult in differentiate the reasons clearly, we hesitated to list the suspicious causes in the study to confuse the result analysis because the causes of deterioration might come from consensus without strong evidence. We only focused on facts in patients' status without speculating on the causes. 

The long-term result in graft failure after heart transplantation was inferior to those without graft failure after heart transplantation, which was reconfirmed when the data was published elsewhere [14]. graft failure after heart transplantation indicated some immunological or perseveration injury during the episodes, and it was reasonable to expect the result. We initially expected that the “early” graft failure after heart transplantation might have the poorer outcome than the “late” graft failure after heart transplantation, but the results showed these two groups were comparable. We also speculated that the “late” graft failure after heart transplantation which needed ECMO support was associated with rejection, which was also related to vasculopathy. Therefore, these groups of patients might be relisted for transplantation earlier before the collapse. Besides, from our previous experience [11], we set up ECMO more aggressively and earlier (72.3% of study group). All these reasons might lead to the similar outcomes between the two groups.

## 5. Limitations

There were some limitations for our present study. First, this is a retrospective study and it ranges for more than 20 years. The improvements in concept and technique might change greatly during this time period. It would definitely change the outcome, and the patients' condition might be more critical now than before. Second, the patient number is not enough for performing a statistic analysis. This is the reason that we only have a risk factor for “success” and “failure” analysis. Third, we hesitated to implant a ventricular assist device (VAD) for patients transplanted, because they had a priority in transplant wait list. It would cause a social and economic conflict. Therefore, we do not have the graft failure after heart transplantation supported with ECMO comparison. 

## 6. Conclusions

Graft failure after heart transplantation requiring mechanical support is still a challenging situation. ECMO can provide 50% survival in the devastating conditions. Early graft failure after heart transplantation and late graft failure after heart transplantation had similar outcomes. Further investigation on the risk factors analysis is required to prevent the graft failure after heart transplantation and improve the outcome. 

## Figures and Tables

**Table 1:** The basic data of the study and the “early” and the “late” graft failures after transplantation (graft failure after heart transplantation).

	Study group	Early	Immediate	Late
Episode, *n*	48	35	30	10
Age, yr (median)	37.7 ± 18.8 (43.3)	36.1 ± 18.6 (42.8)	34.7 ± 18 (35.4)	37.2 ± 20.3 (43.4)
Etiology				
DCMP, *n* (%)	21 (43.8)	15 (42.9)	13 (43.3)	5 (50)
ICMP, *n* (%)	17 (35.4)	14 (40)	11 (36.7)	2 (20)
Other, *n* (%)	10 (20.8)	6 (17.1)	6 (20)	3 (30)
Previous heart surgery	15 (31.3)	14 (40)	10 (33.3)	1 (10)
Pre-Tx ECMO	12 (25%)	12 (34.3)	11 (26.7)	0 (0)*
CPR before ECMO	20 (41.7)	15 (42.9)	12 (40)	4 (40)
ECMO during CPR	10 (20.8)	8 (22.9)	7 (23.3)	1 (10)
IABP use	15 (31.3)	13 (37.1)	12 (40)	2 (20)
Ischemic time, min (median)	178 ± 70 (169)	184 ± 7 (174)	181 ± 73 (171)	189 ± 71 (214)
>120 min, *n* (%)	35 (72.9)	25 (71.4)	21 (70)	7 (70)
>180 min, *n* (%)	23 (47.9)	17 (38.6)	14 (46.7)	6 (60)
>240 min, *n* (%)	11 (22.9)	8 (22.8)	7 (23.3)	3 (30)
ECMO duration, hr (median)	155 ± 145 (103)	148 ± 149 (103)	157 ± 159 (107)	186 ± 147 (143)
Success rate	23 (47.9)	18 (51.4)	15 (50)	5 (50)

*Significant difference for late group with other groups. CPR: cardiopulmonary resuscitation; DCMP: dilated cardiomyopathy; ECMO: extracorporeal membrane oxygenation; ICMP: ischemic cardiomyopathy; IABP: intraaortic balloon pump; Tx: transplantation; yr: year.
